# Decellularized Human Umbilical Artery Used as Nerve Conduit

**DOI:** 10.3390/bioengineering5040100

**Published:** 2018-11-21

**Authors:** Ioanna Gontika, Michalis Katsimpoulas, Efstathios Antoniou, Alkiviadis Kostakis, Catherine Stavropoulos-Giokas, Efstathios Michalopoulos

**Affiliations:** 1 Hellenic Cord Blood Bank, Biomedical Research Foudation Academy of Athens, 4 Soranou Ephessiou Street, 11527 Athens, Greece; igontika@bioacademy.gr; 2 Center of Clinical, Experimental Surgery and Translational Research, Biomedical Research Foundation of the Academy of Athens, 4 Soranou Ephessiou Street, 11527 Athens, Greece; mkatsiboulas@bioacademy.gr (M.K.); akostakis@bioacademy.gr (A.K.); cstavrop@bioacademy.gr (C.S.-G.); 3 Second Department of Propaedeutic Surgery, University of Athens, Medical School, “Laiko” General Hospital 17 Agios Thomas Street, 11527 Athens, Greece; efstathios.antoniou@gmail.com

**Keywords:** umbilical arteries, nerve regeneration, nerve conduit

## Abstract

Treatment of injuries to peripheral nerves after a segmental defect is one of the most challenging surgical problems. Despite advancements in microsurgical techniques, complete recovery of nerve function after repair has not been achieved. The purpose of this study was to evaluate the use of the decellularized human umbilical artery (hUA) as nerve guidance conduit. A segmental peripheral nerve injury was created in 24 Sprague–Dawley rats. The animals were organized into two experimental groups with different forms of repair: decellularized hUA (*n* = 12), and autologous nerve graft (*n* = 12). Sciatic faction index and gastrocnemius muscle values were calculated for functional recovery evaluation. Nerve morphometry was used to analyze nerve regeneration. Results showed that decellularized hUAs after implantation were rich in nerve fibers and characterized by improved Sciatic Functional index (SFI) values. Decellularized hUA may support elongation and bridging of the 10 mm nerve gap.

## 1. Introduction

Peripheral Nervous injuries (PNI) are a global clinical problem, since they significantly affect the quality of life of patients and cause enormous socio-economic burden [1,2,3]. Indicatively, in the United States alone, more than 50,000 peripheral nerve repair surgeries are performed annually [4]. The use of autologous nerve graft is considered as the gold standard procedure for bridging peripheral nerve defects. However, surgical approaches are characterized by several drawbacks. For instance, a secondary surgical procedure, which is often associated with donor site pain and mobility, is needed in order to obtain the nerve graft. Moreover, the sources of neural tissue that can be used as nerve conduits, are particularly limited [5,6,7,8]. Other approaches include the development of three-dimensional scaffold-nerve conduits which can be used for gap bringing between the proximal and distal stump of the nerve tissue. In many cases, nerve conduits also act as cells or growth factors carriers [9,10]. Nerve conduits have been fabricated using different types of material; natural and synthetic, biodegradable and non-biodegradable [11,12]. Natural biological nerve conduits such as vessels (veins and arteries), decellularized nerve [13] and muscle tissue have been widely used to bridge peripheral nerve gap in animal models [14] and also in clinical practice [15,16]. Tissue decellularization offers the possibility to obtain a cell-free, natural extracellular matrix (ECM), characterized by an adequate 3D organization with proper composition to repair different tissues or organs, including peripheral nerves [13].

The human umbilical cord contains two arteries, which can easily be isolated without invasive procedures. Previous reports showed that hUAs retained after decellurization their components such as collagen type I, laminin, and fibronectin [17]. These ECM components are also represented in the ECM of the peripheral nerve [18,19,20]. The aim of this study was to evaluate the use of the decellularized hUA as a nerve guidance conduit in a rat sciatic nerve model.

## 2. Materials and Methods

### 2.1. Collection and Isolation of Human Umbilical Arteries

Human umbilical cords were collected after informed consent from healthy donors. The informed consent was in accordance with Helsinki declaration and approved by the ethical committee of Biomedical Research Foundation Academy of Athens (BRFAA). The cords were stored at 4 °C immediately after birth and the overall storage time until processing did not exceed 24 h. Arteries were isolated from the cords using sterile surgical tools followed by brief rinses in Phosphate Buffer Saline 1× (PBS 1×).

### 2.2. Decellularization of Human Umbilical Arteries

The decellularization of the hUAs was carried out according to previously described protocols, [17]. Briefly, hUAs (*n* = 44, l = 2 cm), were incubated in CHAPS solution (8 mM CHAPS (APPLICHEM, Darmstadt, Germany), 1 M NaCl, and 25 mM EDTA (Ethylenediaminetetraacetic acid) in PBS 1×; (Sigma-Aldrich, Darmstadt, Germany) at pH 8 for 22 h, followed by brief washes in PBS 1×. The hUA were further incubated in SDS solution (1.8 mM SDS (Sigma-Aldrich, Darmstadt, Germany), 1 M NaCl, and 25 mM EDTA in PBS 1×) at pH 7.5 for 24 h, followed by 3 washes for 5 min in PBS 1×, to completely remove the detergent. Finally, the arteries were incubated at 37 °C for 48 h in alpha- Minimal Essential Medium (α-MEM, Gibco Life Technology, Darmstadt Germany), containing 40% (*v/v*) fetal bovine serum (FBS, Gibco Life Technology, Darmstadt, Germany) and 1000 U/mL penicillin-streptomycin (Gibco Life Technology, Darmstadt, Germany). All steps were performed under agitation and sterile conditions.

### 2.3. Histological Evaluation of Decellularized Arteries

Native and decellularized hUAs (*n* = 10) were fixed overnight in 10% *v/v* neutral buffered formalin, embedded in paraffin, cut into 5 μm sections and finally stained with Hematoxylin and Eosin (H&E, Sigma-Aldrich, Darmstadt, Germany) for nuclear material, Masson’s Trichrome (Sigma-Aldrich, Darmstadt, Germany) for collagen content.

### 2.4. Evaluation of Toxicity of Decellularized Umbilical Arteries

#### 2.4.1. Contact Cytotoxicity Assay

The decellularized hUAs (*n* = 10) were cut into 5 × 5 mm and placed in a 24 well culture plate (Orange Scientific, Braine-l’Alleud, Belgium). MSCs (Mesenchymal stem cell) were isolated from Wharton’s Jelly tissue and were seeded into each well at a density of 1 × 10^4^ cells. Then, the samples were incubated at 37 °C in 5% (*v/v*) CO_2_ for 48 h. As positive control group for this assay, SDS was added in MSCs (*n* = 10), and as negative control group MSCs (*n* = 10) were cultured under normal conditions. Morphological examination of seeded cells was performed using brightfield microscope (LEICA DM 1L, Wetzlar, Germany). Images were captured using ΙC Capture 2.2 software. 

#### 2.4.2. ADP/ATP Ratio Assay

Native (*n* = 20) and decellularized hUAs (*n* = 20) were digested using lysis buffer, consisted of 1 mL α-MEM with 1 mg/mL Proteinase K (Sigma-Aldrich, Darmstadt, Germany). The digestion was performed overnight at 56 °C, and the following day the Proteinase K was inactivated at 95 °C for 5 min. The lysates from native and decellularized hUAs were used as culture medium for the evaluation of metabolic activity in MSCs. Then, 1 × 10^3^ MSCs were adhered to each well of 96-wells plate, and the above lysates were added. Specifically, lysate derived from native hUAs was added in 10 wells with adhered MSCs. Lysates derived from decellularized hUAs were added to the next 10 wells with adhered MSCs. MSCs cultured with 1.2 mM SDS (Sigma-Aldrich, Darmstadt, Germany) were used as positive control group. As negative control was used MSCs cultured with standard medium. The culture medium was consisted of α-MEM ((Sigma-Aldrich, Darmstadt, Germany) supplemented with 15% *v/v* FBS (Sigma-Aldrich, Darmstadt, Germany) and 1% *v/v* Penicillin (Sigma-Aldrich, Darmstadt, Germany) and 1% *v/v* Streptomycin (Sigma-Aldrich, Darmstadt, Germany). The 96-well plate was incubated at 37 °C in 5% (*v/v*) CO_2_ for 24 h. Subsequently ADP/ATP ratio assay (Sigma-Aldrich Ratio Assay Kit) was performed according to manufacturer’s instructions.

### 2.5. Animals

Twenty-four male Sprague–Dawley (DS) rats, weighting 250–300 g were randomly divided into two groups (*n* = 12 in each group): The first group was consisted of decellularized hUAs and compared with the second group, which consisted of nerve autograft. The animals were provided by the Animal center of BRFAA and were handled in compliance with the guidelines for the use and care of laboratory animals. Furthermore, all animals were kept in a temperature-controlled room with a 12/12-h light/dark cycle and provided with rodent diet and water ad libitum. The study protocol was approved by the general veterinary directorate and animal health directorate with reference number 2777/26-04-2016 and was accepted by the Bioethics Committee of BRFAA

### 2.6. Surgical Procedure

The animals were anesthetized by isoflurane 3% in 1 L of oxygen. A dorsal gluteal-splitting approach was used to expose and mobilize the right sciatic nerve of each animal. The right sciatic nerve was exposed and a 1 cm gap was made in the mid portion of the nerve. In the nerve autograft group, the removed segment of nerve was oriented at 180° and grafted into the same nerve gap with 6 stiches of prolene 8-0 sutures. In the umbilical artery group, a 1.5 cm artery was grafted into the gap. Both proximal and distal stump were inserted about 2–3 mm from the ends of the artery graft and four stiches were performed in each stump. The manipulations of the nerves were made under an operational microscope.

### 2.7. Sciatic Functional Index (SFI)

The functional condition of the animals was assessed with the estimation of SFI, according to Bain et al. Formula [21]:

$$\;-38.3\frac{EPL-NPL}{NPL}+109.5\frac{ETS-NTS}{NTS}+13.3\frac{EIT-NIT}{NIT}-8.8.\;$$

Walk track analysis was performed at pre-operative, and at the 4th and 12th week after the surgery [22]. The rats’ hind feet were painted with ink and the animals were placed in a walking pathway to walk down the track, leaving their footprint. Footprints from the experimental (E) and contralateral normal (N) sides were analyzed by measuring the lengths of the third toe to heel (PL), the first toe to the fifth toe (TS), and the second toe to the fourth toe (IT). Index values close to 0 indicated normal function and values close to −100 represented loss of function.

### 2.8. Nerve Graft Harvested Tissue

Twelve weeks postoperatively, the regenerated sciatic nerves were harvested. The midportion of the graft *n* = 6 from each group were fixed with 10% neutral buffered formalin solution, for immunohistochemical analysis. In addition, the other *n* = 6 from each group were fixed with 2.5% glutaraldehyde in 0.1 M phosphate buffer for morphometric analysis. Transverse sections were cut both in immunochemistry and in morphometry. The sections which were analyzed in this assay, were 5 mm distal from the side of proximal lesion.

### 2.9. Nerve Immunohistochemistry

Grafts were fixed in 10% *v/v* neutral buffer formalin solution (Sigma-Aldrich, Darmstadt, Germany) paraffin embedded and sectioned. Then, the slides were deparafinnized, rehydrated and blocked. Dako Envision Flex kit was used for the immunohistochemistry assay according to manufacturer’s instructions (Dako, Agilent, Glostrup, Denmark). Briefly, nerve graft sections were incubated at 4 °C over night with rabbit anti-neurofilament 200 (nf 200) antibody (1:80, Sigma, St. Louis, MO, USA) to identify axons and S100 antibody (1:100, Sigma, St. Louis, MO, USA) to identify Schwann cells. Briefly, washes were performed, and addition of horseradish peroxidase (HRP) conjugated with goat secondary antibody against rabbit and mouse was performed. The slides were incubated at Room Temperature (RT) for 45 min. Finally, 3′3 diaminobenzidine (DAB) was added to the slides. Slides were visualized by light microscopy and images were acquired with ΙC Capture 2.2 software and processed with imageJ software version 1.52g.

### 2.10. Morphometric Analysis of Nerve

The midportion of the graft n = 6 from each group was fixed with 2.5% *v/v* glutaraldehyde (Sigma-Aldrich, Darmstadt, Germany) in 0.1 M phosphate buffer (pH 7.4) for 48 h at room temperature and post-fixed with 1% osmium tetroxide (Sigma-Aldrich, Darmstadt, Germany). The nerve specimens were embedded in epoxy resin, cut into 1-μm, semi-thin sections with an ultramicrotome and stained with 1% toluidine blue (Sigma-Aldrich, Darmstadt, Germany) for light microscopy. Images were digitized with a charge-coupled device camera and analyzed with standard image processing at a magnification of ×1000. Ten random fields from each semi-thin section were analyzed with imaging software (IMARIS 8, Bitplane, Zurich, Switzerland). The sample area was chosen in a systemic, uniform, random manner ensuring that all locations in the nerve cross-section were equally represented. The number of nerve fibers was counted, followed by estimation of mean fiber area and density of myelinated nerve fibers (fibers/μm^2^) were determined [7].

### 2.11. Gastrocnemius Muscle Histology and Muscle Weight Ratio

The gastrocnemius muscle was weighted on an analytical balance immediately after removal from the animals from both sides, normal and experimental, and muscle weight ratio was calculated. Then, the middle part of the muscles was cut and put in a 10% natural formalin solution overnight. The muscles were embedded in paraffin and cut on a microtome into transverse sections at 5 μm, which were subjected to H&E staining followed by observation under light microscope.

### 2.12. Statistical Analysis

Data was expressed as mean ± standard deviation (SD) and statistical analyses, performed using Graph Pad Prism 6 software (GraphPad Software, San Diego, CA, USA). All data was analyzed with non-parametric student’s test, except ADT/ATP essay data that was analyzed with Kruskal–Wallis test and the statically significance level was defined at *p* < 0.05.

## 3. Results

### 3.1. Histological Analysis

**Figure 1:**
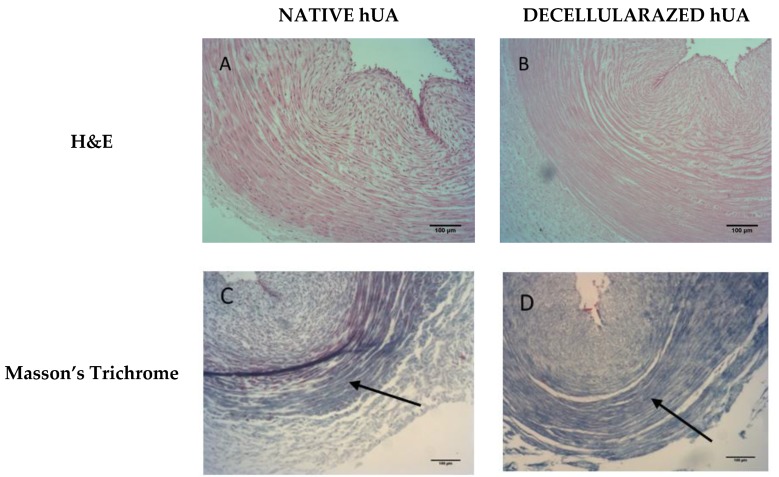
Native and Decellularized human umbilical artery stained with H&E (Hematoxylin and eosin stain), Masson’s Trichrome. Native hUA with (**A**) H&E, (**C**) Masson’s Trichrome. Decellularized hUA (**B**) H&E, (**D**) Masson’s Trichrome. Black arrows were indicated the collagen orientation. Original magnification 10×, scale bars 100 μm.

Histological analysis was performed in order to evaluate the impact of decellularization procedure in hUAs. Specifically, H&E showed the preservation of ECM, while the cellular populations were totally absent. Furthermore, Masson’s Trichrome staining revealed the presence of properly oriented collagens in decellularized hUA (Figure 1).

### 3.2. Cytotoxicity Tolerance of the Decellularized hUA

**Figure 2:**
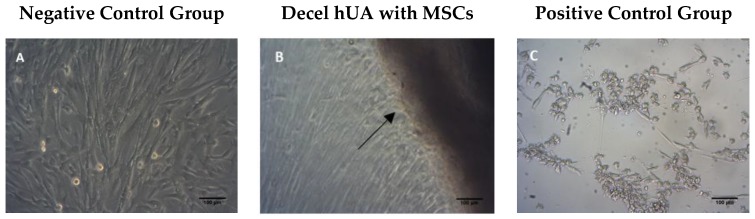
Contact cytotoxicity assay. (**A**) MSCs cultured under normal conditions (negative control group); (**B**) MSCs were co-cultured with decellularized hUA, (**C**) MSCs with SDS (positive control group). Black arrows indicate the contact of MSCs with the segments of decellularized hUA. Original magnification 10×, scale bars 100 μm.

**Figure 3:**
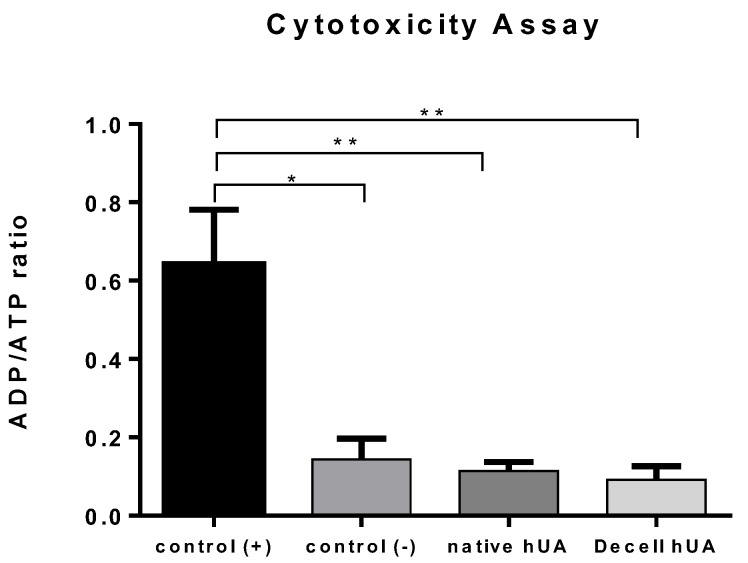
ADP/ATP ratio assay. Low ADP/ATP ratio values indicates that the cells preserved their proliferation capacity. * *p* < 0.05, ** *p* < 0.001.

Contact cytotoxicity assay results showed that MSCs were expanded and attached successfully to the decellularized hUA segments in 96 well-plates after 48 h of incubation (Figure 2). Moreover, the cells were characterized by the same morphology as the MSCs from negative control group, indicating no cytotoxicity. These findings were further confirmed by determination of ADP/ATP ratio. Specifically, ADP/ATP ratio values were similar between, native, decellularized hUA samples and negative control group. Statistically significant difference was observed only between positive control group, native (*p* < 0.001) and decellularized (*p* < 0.001) hUA samples (Figure 3).

### 3.3. Macroscopic Examination of the Experimental Sciatic Nerve In Situ

**Figure 4:**
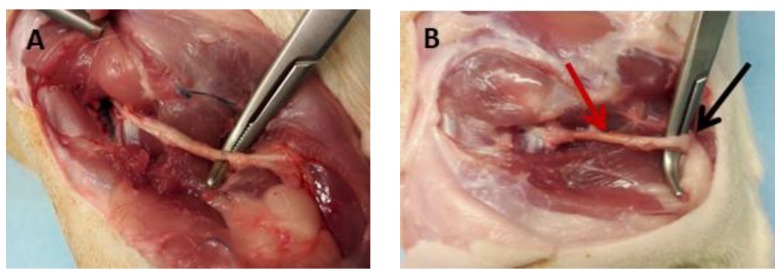
(**A**) autologous nerve graft; (**B**) hUA graft. The red arrow indicates the graft and the black arrow indicates the nerve tissue.

All animals survived until the end of the experiment and were in good health as indicated by visual inspection. No signs of self-injury were observed in the operated limb. After euthanasia, the implanted nerve conduits derived either from autograft or decellularized group were visually checked. Regenerated nerves were passed through the nerve conduits from both experimental procedures and bridged successfully the 10 mm gap. No evidence of inflammation was observed in both groups. Furthermore, the thickness of the regenerated nerves was similar in animals of both experimental groups (Figure 4).

### 3.4. Motor Function Assessment

The recovery of motor function was assessed by calculating the SFI pre-operative animals, after 4 and 12 weeks. The SFI in all rats prior to surgery was within normal range. SFI values were −10.07 ± 3.38 at the autograft group and −8.26 ± 5.1 at the hUA group without a statistically significant difference. The values of the SFI were reduced at the first post-operative evaluation at week 4 for both groups. SFI values of autograft group and decellularized group was −87.36 ± 6.27 and −80.89 ± 9.22, respectively. No statistically significant difference was observed between the above groups (*p* > 0.05). At 12 weeks, the values of the autograft group showed better improvement than the ones of the hUA group (*p* = 0.0013). Nevertheless, none of these two groups approached the normal values of SFI (Table 1).

### 3.5. Immunohistochemical Detection of Neurofilaments and Schwann Cells

**Figure 5:**
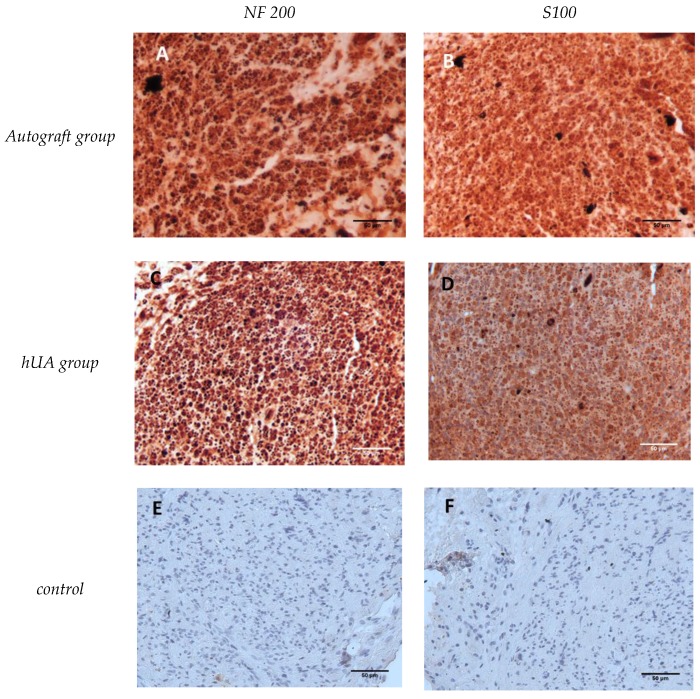
Immunohistochemical results 12 weeks after implantation. (**A**,**C**) transverse sections of, autograft group and hUA group respectively, stained with NF200 antibody; (**B**,**D**) transverse sections of autograft group and hUA group respectively, stained with S100; (**E**,**F**) transverse sections without primary antibody served as a negative control. Original magnification 40× scale bar 50μm.

The transversal section from each group of nerve grafts were stained with anti-NF 200 and anti-S100 antibody for evaluating the axon regeneration. Positive expression of NF200 and S100 detected in all sections (Figure 5A–D).

### 3.6. Morphometric Analysis

**Figure 6:**
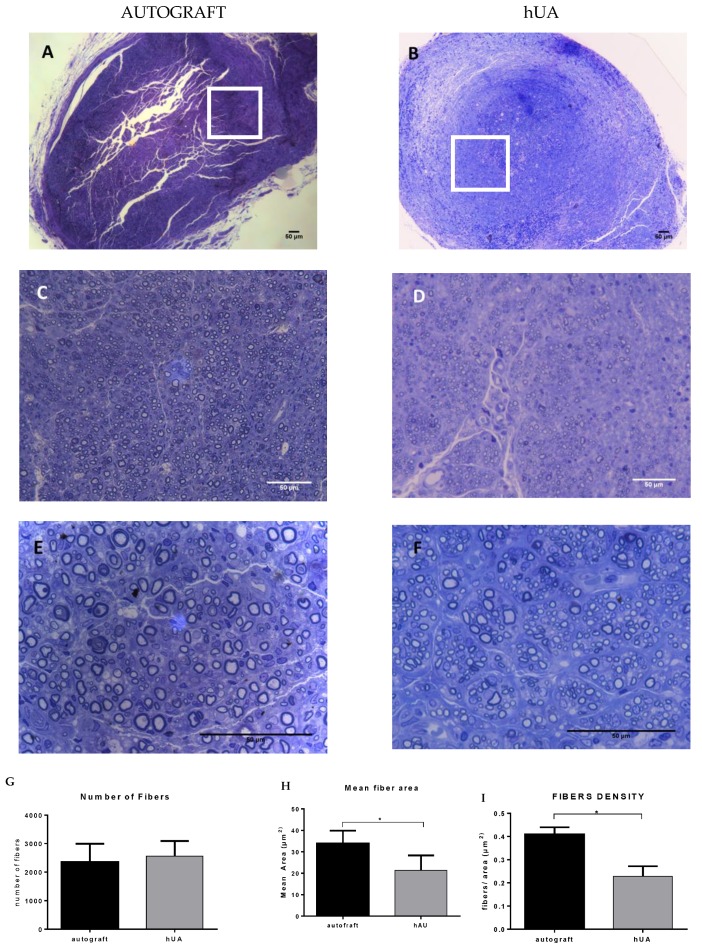
Morphometric analysis of regenerated sciatic nerve (**A**,**C**,**F**) toluidine blue stained, middle portion of the autograft after 12 weeks at different magnifications. 10, 40 and 100× respectively; (**B**,**D**,**E**) toluidine blue stained, middle portion of the hUA graft after 12 weeks at different magnifications. 10, 40 and 100×, respectively. The white boxes in (**A**,**B**) highlighted the area that was magnified and presented in the following images (**C**,**D**,**E**,**F**) scale bar 50 μm; (**G**) number of fibers; (**H**) mean fiber area; (**I**) fiber density was evaluated and compared to statistical analysis * *p* < 0.05.

Morphometric analysis was performed in the middle portion of the grafts at week 12. Regenerated myelinated nerve fibers, different in sizes, were observed in each group (Figure 2A,B). The number of nerve fibers between the two groups did not present a statistically significant difference (Figure 6C), but the nerve fibers areas had a wide distribution range (9.87–21.25 μm^2^). The area of the hUA group was significantly smaller than that of the autograft group (*p* = 0.0073, Figure 2D). This trend was also reflected in the density of the nerve fibers (*p* < 0.0001, Figure 6E).

### 3.7. Gastrocnemius Muscle Histology and Muscle Weight Ratio

**Figure 7:**
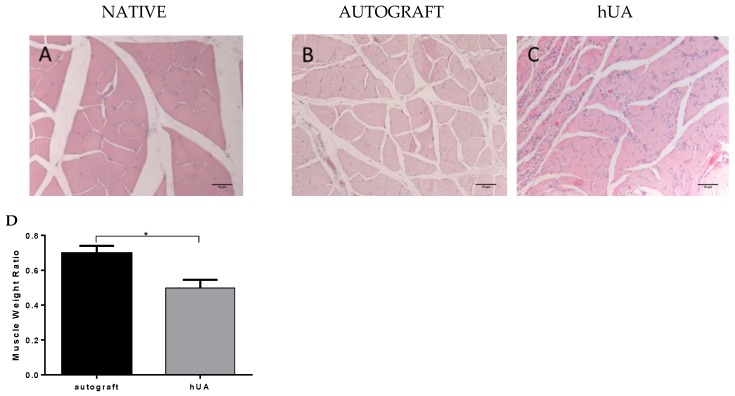
Gastrocnemius muscle histology and weight ratio. (**A**–**C**) gastrocnemius muscle transverse section stained with H&E from normal, autograft group and hUA group, respectively. Original magnification 40×; (**D**) muscle weight ratio was evaluated and compared by statistically analysis * *p* < 0.05.

In both experimental groups, gastrocnemius muscle showed intense atrophy, while the autograft group presented less atrophy, when compared to the decellularized hUA group. The ratio of muscle mass retention of autologous and decellularized group was 0.55 ± 0.10 and 0.33 ± 0.3, respectively. (Figure 7D). The difference between the two groups is statistically significant (*p* < 0.001). These results were also supported by the histological evaluation. More specifically, in the autograft group, fibers appeared polygonal with sub-sarcolemmal localization of their nuclei and minimal growth of connective tissue. In the hUA group, the muscle fibers formed small groups of atrophic fibers and more fibrous connective tissues among muscle bundles. In addition, decellularized hUA presented an increased number of cell nuclei when compared to autograft groups (Figure 7A–C).

## 4. Discussion

Peripheral nerve injuries are very common worldwide, and there is no easily available treatment. Decellularized grafts could be used as an alternative source for nerve conduits. These grafts are characterized by reduced antigenicity and could be a promising therapeutic strategy, when no autologous tissues are available [23].

Different types of decellularized tissues such as nerves and arteries have been used for reconstruction of transected peripheral nerve and showed promising results [24,25]. In this context, hUAs, which can be efficiently isolated from human umbilical cords, a material that is discarded after the gestation, may be good candidates for peripheral nerve reconstruction. The hUA is composed of a complex ECM, which apparently includes collagen, fibronectin, laminin and proteoglycans [17,20]. These proteins, especially laminin, promote neurite and enhance nerve cells adhesion, proliferation and differentiation, thus helping to direct growth cone neurite [26,27]. Due to their importance during the development and the regeneration of the sensory nervous system, laminin, fibronectin and collagen have been successfully used as substrates of tissue culture plastic and poly-3-hydroxybutyrate mats to enhance Schwann cell (SC) response [28]. Furthermore, after the decellularization procedure, the proteoglycans significantly reduced as has been confirmed by others [17]. In this context, chondroitin sulfate, which has a negative impact on nerve guidance and regeneration, can be removed efficiently by the decellularization approach.

Histological analysis indicated the absence of cellular and nuclear residues. Additionally, the structural proteins of the ECM, such as collagen, were preserved when compared to native arteries. After the decellularization of the hUAs, the ATP assay was performed and MSCs were co-cultured within 5 × 5 mm patches of decellularized tissue. As it was expected, the tissue supported the cell attachment and the ADP/ATP assay confirmed the maintenance of proliferation capacity of the cells. MSCs were used for this assay because they are characterized by multilineage differentiation potential. Previous studies have shown that MSCs can differentiate efficiently to neuronal like cells. In addition, future experiments will involve the repopulation of the decellularized hUA with MSCs, implantation in the rat sciatic model, and final evaluation of the function between decellularized and repopulated hUAs. For this purpose, we used MSCs and not neural cells for the cytotoxicity assay. After implantation, artery conduits supported the regeneration sciatic nerves and no inflammatory response was observed.

Previous studies have shown that nerve-conduits have better outcomes, which were similar to autograft results, or even better when they are loaded with different types of cells like adipose-derived stem cells, olfactory cells, Schwann cells, neurotrophic factors or platelet rich plasma [29,30,31,32,33,34]. In this study, hUA was used alone as an initial step to find out whether it can support elongation of the nerve fibers.

Walk track analysis was performed by estimation of SFI for evaluation of the motor function in animals. Our results demonstrated that neither the autologous nor the decellularized hUA graft restored the SFI close to pre-operative values. Nevertheless, a better functional outcome was observed at the autograft graft group (−51.35). Yeong Kim et al. [32] had similar results on the same week (12th) of SFI evaluation. On the contrary, in other studies, better outcomes were obtained after repairing nerve gaps with autologous grafts (−23.4) [35].

Morphometric and immunohistochemical analysis confirmed the elongation of the nerve fibers and also that the hUA was recellularized and remodeled successfully by the animals.

Immunohistochemical analysis showed positive expression positive of NF200 and S100 in both experimental groups. These findings indicated that decellularized hUA allows the migration of Schwann cells and elongation of the fibers through the umbilical artery tissue.

Morphometric analysis showed the number of fibers between the two experimental groups did not present any statistically significant difference (*p* = 0.563). However, the area and density of the nerve fibers were higher in the autograft group compared to decellularized hUA. These findings may suggest that the nerve fibers at the hUA group were still in a pre-mature stage of [36]. Another parameter to evaluate the re-innervation in the sciatic nerve lesion model is the gastrocnemius muscle weight ratio. When a muscle is denervated, it shifts to degradation, which leads to weight loss [36]. In both experimental groups, the gastrocnemius muscle showed atrophy. The hUA group showed higher atrophy than the autograft group and more cell n. However, normal and smaller muscle fibers co-existed at the hUA group as it was observed at the histological image. However, more cell nuclei were still observed at the hUA group. This can be explained partially by the fact that muscle atrophy was established in decellularized hUA group. Further clarification could be performed by immunohistochemistry for CD11b (macrophage marker) and pro collagen beta 1 (fibroblast markers) [37].

## 5. Conclusions

In conclusion, this study showed that the decellularized hUA could support the nerve regeneration and could allow the reinnervation of the target organ. Further research in decellularized hUA is needed in order to be used as nerve conduits. Glycosaminoglycans (GAGs) such as chondroitin sulfate, which are important components of hUA, must be properly identified.

In future studies, the decellularized hUAs could be combined with different cell populations or neurotrophic factors, in order to obtain better outcomes, thus bringing them one step closer to clinical application.

## Figures and Tables

**Table 1:** Values of SFI ± SD.

	Pre-Operative	4 Weeks	12 Weeks
Autograft group	−10.07 ± 3,38	−87.36 ± 6,27	−51.35 ± 7.84
hUA group	−8.26 ± 5,1	−80.89 ± 9,22	−70.56 ± 15.38
